# Post-Growth Dynamics and Growth Modeling of Organic Semiconductor Thin Films

**DOI:** 10.1021/acs.langmuir.2c03066

**Published:** 2023-02-22

**Authors:** Alice Pancaldi, Luisa Raimondo, Alessandro Minotto, Adele Sassella

**Affiliations:** Department of Materials Science, University of Milano-Bicocca, via R. Cozzi 55, 20125 Milano, Italy

## Abstract

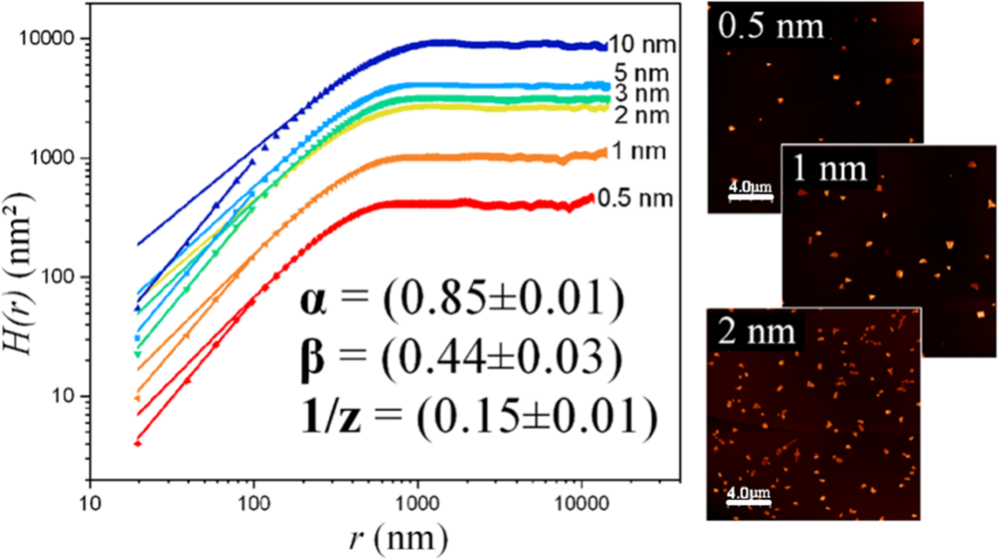

The ability to control the properties of organic thin films is crucial for obtaining highly performant thin-film devices. However, thin films may experience post-growth processes, even when the most sophisticated and controlled growth techniques such as organic molecular beam epitaxy (OMBE) are used. Such processes can modify the film structure and morphology and, thus, the film properties ultimately affecting device performances. For this reason, probing the occurrence of post-growth evolution is essential. Equally importantly, the processes responsible for this evolution should be addressed in view of finding a strategy to control and, possibly, leverage them for driving film properties. Here, nickel-tetraphenylporphyrin (NiTPP) thin films grown by OMBE on highly oriented pyrolytic graphite (HOPG) are selected as an exemplary system exhibiting a remarkable post-growth morphology evolution consistent with Ostwald-like ripening. To quantitatively describe the growth, the height–height correlation function (HHCF) analysis of the atomic force microscopy (AFM) images is carried out, clarifying the role of the post-growth evolution as an integral part of the whole growth process. The set of scaling exponents obtained confirms that the growth is mainly driven by diffusion combined with the presence of step-edge barriers, in agreement with the observed ripening phenomenon. Finally, the results together with the overall approach adopted demonstrate the reliability of the HHCF analysis in systems displaying post-growth evolution.

## Introduction

In the last few decades, organic semiconductors have been the focus of many research activities in the field of optoelectronics, thanks to their nowadays well-established advantages compared to inorganic semiconductors.^1^

Organic electronics typically relies on thin films used as active layers in devices,^2,3^ whose performances are found to be highly dependent on the film structure and morphology, directly dictating the optoelectronic properties.^4,5^ For a successful integration in devices, control over the film growth is, therefore, crucial.^6^ One of the best techniques to obtain thin films with high control on structure and morphology is organic molecular beam epitaxy (OMBE), which allows fine-tuning of the growth parameters.^7−11^ Nonetheless, even films grown via OMBE may experience a post-growth evolution in a controlled environment and/or in ambient conditions, through different processes such as wetting,^12^ dewetting,^13^ or ripening,^14−16^ often characterized by a change in the morphology and/or structure,^17−23^ which may affect device performance and long-term stability. It is, therefore, important to determine the occurrence of such processes and characterize them to find a strategy to control them. Several factors may contribute to the possible post-growth evolution in thin films, such as the type of substrate, the substrate surface energy, the shape of the molecule chosen, and the film thickness. Another relevant factor is represented by the environmental conditions (vacuum, inert, or ambient atmospheres) which could also play a fundamental role.^24^ In any case, evolution comes from a combination of all of the mentioned elements. Interestingly, the possibility of achieving a fine control on post-growth phenomena with a proper sample preparation protocol has been recently demonstrated.^17,25−28^

One of the easiest and most useful techniques to probe and monitor film morphology is atomic force microscopy (AFM). A judicious use of suitable methods for the analysis of AFM images, such as fractal-based ones, also enables the modeling of the growth process.^29−32^ In particular, the theoretical approach based on the modeling of height fluctuations, applied to experimental data through the height–height correlation function (HHCF),^33−35^ is an established powerful method that provides a description of the growth process through a set of scaling exponents. These exponents characterize and quantify the surface spatial correlation, together with the temporal evolution of the lateral correlation length and the roughness. Although this method was originally designed to describe self-affine surfaces, its validity has already been extensively demonstrated also for non-self-affine surfaces, e.g., mound-like surfaces, which possess a characteristic length.^36^ This approach, commonly applied to inorganic and stable films, has also been proposed as a successful strategy to get a description of organic film growth,^12,31,32,37−40^ in most cases displaying good post-growth stability.

In the present work, a detailed investigation of the growth process of organic semiconductor thin films affected by post-growth phenomena is carried out by the careful use of HHCF. Nickel-tetraphenylporphyrin (NiTPP)^41−44^ deposited via OMBE on highly oriented pyrolytic graphite (HOPG) is selected here as a paradigmatic example of a system showing evident post-growth evolution on a long time scale (on the order of weeks), as observed via ex situ AFM monitoring. Building on these findings, a quantitative description of the whole growth process is obtained by applying the HHCF analysis, assuming that the post-growth evolution is an integral part of the growth process itself.

## Experimental Details

Polycrystalline NiTPP powder is purchased from Sigma–Aldrich and used to grow thin films on *zyb*-grade HOPG (Union Carbide Corporation) substrates, mechanically exfoliated by adhesive tape and kept at room temperature during film growth, carried out by means of OMBE. The OMBE apparatus works under high vacuum, with a base pressure of ∼10^–7^ Torr, and is equipped with an effusion cell kept at 307 °C. The nominal thickness of the growing films is monitored with a quartz microbalance. A set of six samples with thickness from 0.5 to 5 nm is deposited, keeping the deposition rate constant (1.6 ± 0.2 Å/min). Right after the deposition, the samples are maintained in a dynamic vacuum condition (∼10^–2^ Torr) for about 3 h, which is the minimum time needed for the apparatus to cool down, and then extracted for characterization. The surface morphology of each sample is then monitored ex situ (in ambient conditions) by AFM with a Nanoscope V MultiMode (Bruker) instrument. An important parameter for the description of the monitoring is the aging time, hereinafter labeled as *t*_a_, which is the time elapsed since the sample extraction from the vacuum chamber. AFM images are collected in intermittent-contact mode in air with an AS-130 (“J”) piezoelectric scanner (maximum scan size: 125 × 125 μm^2^; vertical range: 5 μm) and R-TESP 300 Sb-doped Si probes (nominal resonance frequency: 300 kHz; nominal elastic constant: 40 N/m). All of the AFM images are analyzed by using WSxM^45^ and Gwyddion.^46^ To get good statistical reliability, for each sample, up to ten AFM images, collected in different zones on the surface, are considered. Finally, to carry out the HHCF analysis, the data sets extracted from each image (20 × 20 μm^2^) are averaged.

## Results and Discussion

### Film Morphology

**Figure 1:**
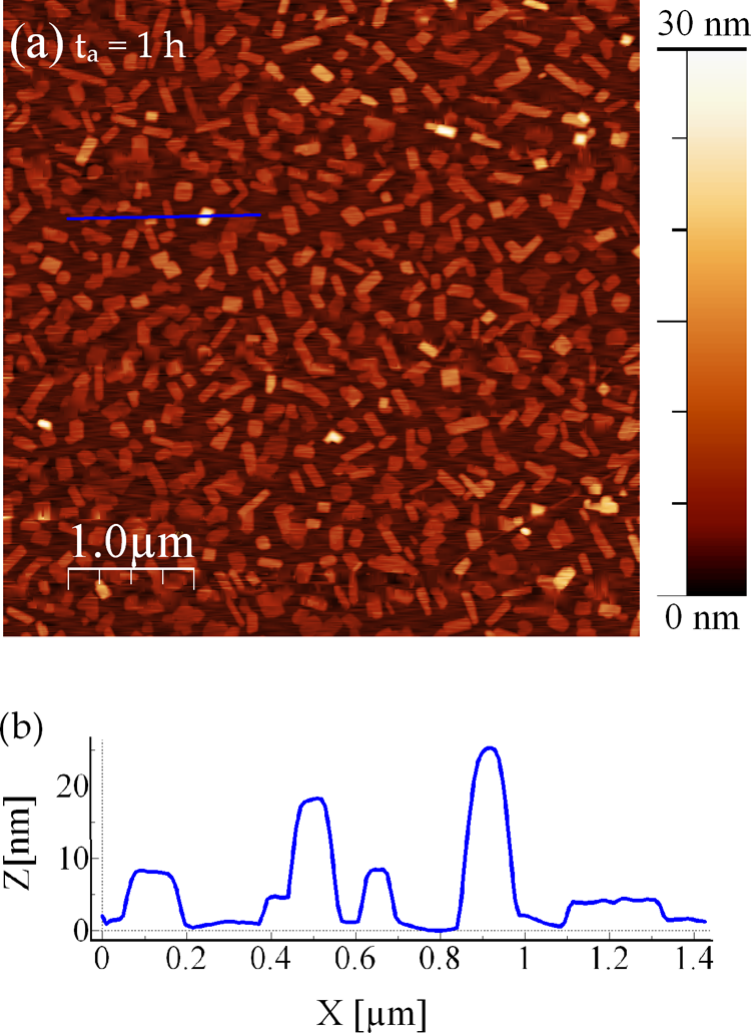
(a) 5 × 5 μm^2^ AFM image of a 2 nm thick NiTPP film, taken at *t*_a_ = 1 h. The profile along the blue line is highlighted in (b).

In Figure 1a, the morphology of a 2 nm thick film of NiTPP at *t*_a_ = 1 h is reported: the film is rather uniform and composed of polygonal-shaped islands with a mean lateral size on the order of tens of nm; it is characterized by a 30% coverage and a total volume of about 10^8^ nm^3^ (over the scanned 5 × 5 μm^2^ area). Most of the islands, at this early stage of aging, have a height ranging from a few nm to around 20 nm and frequently show flat top surfaces, as illustrated in the profile reported in Figure 1b. The neat edges and flat top surfaces suggest that the islands are crystalline.

**Figure 2:**
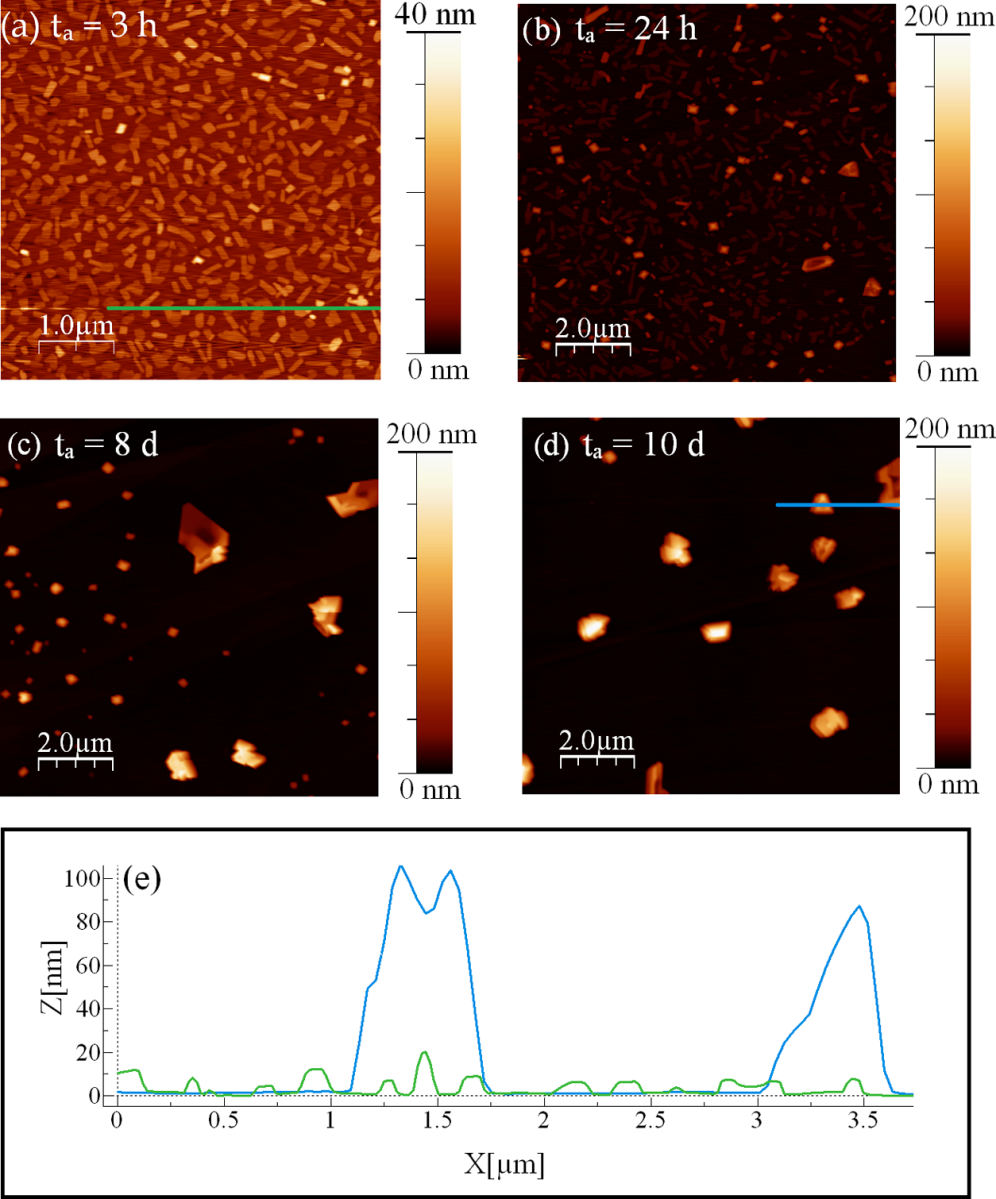
AFM height images of a 2 nm NiTPP thin film on HOPG representative of different *t*_*a*_: (a) 3 h, (b) 24 h, (c) 8 days, and (d) 10 days. For each image, the height scale and the image size are set to improve the visibility of the islands. In panel (e), the profiles along the lines highlighted in the images (a) (green line) and (d) (blue line) are reported.

The film morphology is monitored every 0.5 h for the first 3 h, then after 24 h, and at least once a day for 10 days. In Figure 2, a set of AFM images of the same 2 nm thick film as that in Figure 1 is reported, representative of various *t*_a_.

In Figure 2a (*t*_a_ = 3 h), the film shows a very similar morphology as seen in Figure 1a, with a total film volume of about 10^8^ nm^3^ (over the 5 × 5 μm^2^ area); the majority of the islands are flat and with a height less than 20 nm (see the green profile in Figure 2e). At longer *t*_a_, a gradual post-growth evolution is noticeable, and the volume is conserved. Indeed, in Figure 2b (*t*_a_ = 24 h), the substrate coverage is reduced to 20%, and two different families of islands are distinguishable: the first one is composed of flat and polygonal-shaped islands, very similar to those observed in Figures 1a and 2a, and the other one consists of bigger islands reaching heights of 50–70 nm and lateral sizes ranging from hundreds of nm to almost 1 μm. Such an evolution goes on. In Figure 2c, collected at *t*_a_ = 8 days, the first family of islands is still present, but drastically reduced in number, while the total volume is maintained. In contrast, the islands of the second family have grown further, reaching lateral sizes of 0.5–2 μm and heights from 60 to 100 nm. These islands frequently show irregular top surfaces, with edges higher than the central region. Furthermore, at this stage, the coverage is reduced to ∼6%. Finally, Figure 2d shows the film after *t*_a_ = 10 days: only the biggest islands are observed, while the other ones have disappeared after gradually reducing in number with *t*_a_. The final coverage is ∼5% and the total volume is still conserved, as expected. As suggested by the profile in Figure 2e (blue line), the islands at this stage are characterized by heights above 80 nm and irregular surfaces, very different from the initial flat ones (green line). It is also important to note that since no further evolution is detected after *t*_a_ = 10 days, at this stage of aging, the film is considered in a steady state.

Monitoring of all films with different thicknesses shows the same post-growth evolution, with the only difference that, by increasing the nominal thickness, the coverage is higher and the steady state is reached after longer *t*_a_, up to 14 days (Figures S1–S3 in the SI). In all of the films, the morphology is therefore seen to evolve via an Ostwald-like ripening process, whereby a mass redistribution leads to the growth in the size of some of the islands at the expense of the smaller and less stable ones, possibly with a different structure. Notably, an analogous behavior was also observed in previous studies of organic molecules (including porphyrins) deposited on different substrates.^14−17,47^

### Analysis and Modeling

To obtain a model of the growth dynamics of the NiTPP films on HOPG, the AFM images are analyzed by means of the HHCF.

The HHCF, *H*(*r*,*t*), is defined as the mean square of height difference in the AFM image of the film between two surface positions separated by a distance *r*
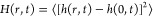
1where *h*(*r*,*t*) and *h*(0,*t*) are the height values at the two positions for a specific deposition time *t* (note that for the samples presented here the deposition rate was constant; therefore, *t* can also be interpreted as the nominal thickness). The average in eq 1 is calculated over all of the pairs of points and therefore heights in the AFM images.

The correlation curves obtained can be, then, fitted to the following phenomenological function^36^
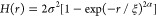
2where σ is defined as the long-range surface roughness or root-mean-square (RMS) roughness, ξ is the lateral correlation length, and α is a characteristic exponent that describes the local roughness of the film. A visual description of the physical meaning of σ and α for different roughness values is reported in Figure S4 in SI.

Although the HHCF analysis was originally proposed to describe self-affine surfaces, it has been successfully adapted to mound-like surfaces,^36,48^ which possess a characteristic length scale, by slightly modifying eq 2. In the case of island-like morphology, like the one observed in our NiTPP films (Figure 2), it is reasonable to consider such a modified equation,^12,49^ namely
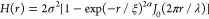
3In eq 3, the presence of the islands on the surface is accounted for by an oscillatory factor, that is, the Bessel function *J*_0_, where λ stands for the average separation of the islands. In this case, ξ can be considered as the average size of the island^36,48^ and, therefore, must satisfy the relation ξ ≤ λ (the mounds are separated by at least their size). Further support for the choice of eq 3 for the present study comes from the application of the fast Fourier transform to AFM images (see Figure S5).

Crucially, from the HHCF analysis, a set of parameters, namely, α, β, and 1/*z*, can be extracted. From their values, it is possible to get insights into the growth process of the films. The first parameter α, present in eqs 2 and 3, is usually found in the range 0 ≤ α ≤ 1 and describes the local height fluctuation across the image: when α ∼ 0, the film is very rough in the local scale, and if α ∼ 1, the film is smooth. However, it is known^48^ that the use of eq 3 to directly obtain α leads to an overestimation of its value; for this reason, α is usually extracted from a linear fit of the log–log plot of *H*(*r*) in the range *r* ≪ ξ (as schematically illustrated in Figure S4), where the following simplified expression for the HHCF can be used
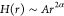
4in which *A* is a constant. The linear dependence found in the range *r* ≪ ξ indicates that on length scales much smaller than λ, a mounded surface appears self-affine because of the absence of a characteristic length scale smaller than λ in that range.^34,36^

The other two important parameters β and 1/*z* can be derived from σ and ξ directly obtained by fitting the correlation curves to eq 3. Namely, σ and ξ can be expressed as power functions of *t*(34,36)
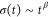
5
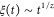
6where β and 1/*z* appear as scaling exponents.

The exponent β, typically defined as the scaling growth exponent, characterizes the type of growth process, and defines, for example, the type of roughening or smoothing. In the literature, different values of β are encountered for organic films depending on the deposition rate, substrate temperature, and type of substrate.^8,11,39^ The value β = 0.5 corresponds to the stochastic limit for the kinetic growth, while β > 0.5 is related to the phenomenon of “rapid roughening”, whereby the roughness σ increases faster than the natural deposition limit of the stochastic roughening.^34,50^ The exponent 1/*z*, also known as the dynamic exponent, is related to the evolution of the correlation length. Specifically, 1/*z* is sensitive to local effects, such as the occurrence of diffusion. Together, the exponents β and 1/*z* describe and quantify the evolution of the islands during growth.

An important consideration must be made on the samples investigated here before feeding the corresponding AFM data into eq 3. Namely, as discussed in the previous section, NiTPP films grown on HOPG experience evident post-growth evolution, reaching the steady-state condition at different *t*_a_ for different film thicknesses. Therefore, at *t*_a_ ∼ 0, i.e., right after the extraction, the stage of the post-growth evolution for each sample with different thicknesses is obviously different. For this reason, the HHCF analysis should not be applied to images at *t*_a_ ∼ 0 of samples with different NiTPP nominal thicknesses. As a confirmation, the use of those images leads to unintelligible results (Figure S6). On the contrary, the AFM images of samples in their “steady state” (Figure S3) should be analyzed, therefore, considering the observed post-growth evolution as an integral part of the growth process.

**Figure 3:**
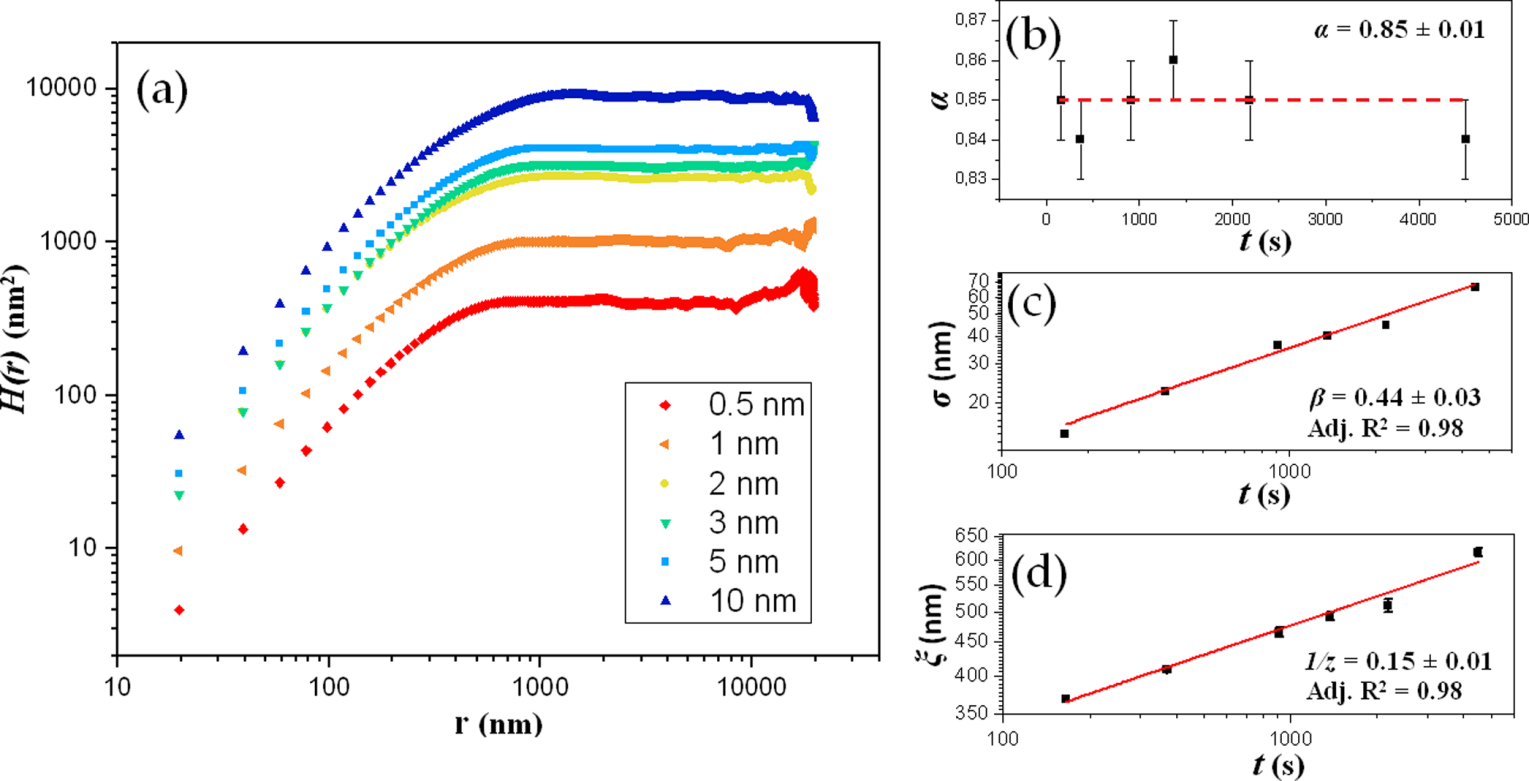
(a) Averaged *H*(*r*) data sets for the six samples with various thicknesses, from 0.5 to 10 nm. (b) Values of α as a function of *t*; the mean value of α (average of all α values corresponding to the different *t*) is also shown as a dashed line. (c) log–log variation of σ with respect to *t* (error bars smaller than symbols) and β value extracted by fitting the σ(*t*) data to eq 5. (d) log–log variation of ξ with respect to *t* and 1/*z* value extracted by fitting the ξ(*t*) data to eq 6. Adjusted *R*^2^ values are reported in panels (c) and (d).

In Figure 3, the *H*(*r*) curves obtained via eq 3 from the “steady state” for all of the samples are reported (Figure 3a), together with the values of the scaling exponents obtained from the analysis (Figure 3b–d). The complete fits of HHCF data sets are reported in Figure S7.

As shown in Figure 3a, each *H*(*r*) curve clearly shows the linear dependence on *r* in the range *r* ≪ ξ in the log–log plot, thus allowing the extraction of α as a function of *t* through eq 4 (Figure 3b). Then, by fitting the whole *H*(*r*) data set using eq 3, σ and ξ versus *t* are extracted as shown in Figure 3c,d, respectively. Finally, through eqs 5 and 6, the same σ and ξ values can be used to obtain the values of the exponents β and 1/*z*. Hence, from this analysis, the characteristic exponents found for the growth of NiTPP on HOPG considering the whole set of samples are α = 0.85 ± 0.01, β = 0.44 ± 0.03, and 1/*z* = 0.15 ± 0.01. Looking more closely at the values of the parameters obtained from the HHCF analysis, it can be observed that, similarly to what is observed in other organic thin films,^32,37,38^ α falls in the range between 0 and 1, as expected, closer to 1: the islands at their final stage are, indeed, characterized by top surfaces with frequently observed raised edges or pits in the centers, but they are locally smooth. In addition, since the substrate coverage of all of the films is rather low (around 5%), the high value of α might also be due to the contribution of the free substrate surface, indeed very flat.

The value of the second exponent β is found to be right below the stochastic deposition limit (0.5), in accordance with the observed roughening process also found in other organic thin films.^12,32,38^

Finally, the value of the third exponent 1/*z* is found to be lower than 0.2: the increase of ξ with *t*, observed in Figure 3d, indicates that the lateral size of the islands increases with the amount of material deposited onto the substrate.

It is worth highlighting that the values of these scaling exponents are very similar to those reported in the literature^32,37^ for roughening of the metal phthalocyanine thin films grown on inorganic surfaces. This agreement is indeed particularly meaningful, given the similarities between porphyrins and phthalocyanines.

**Figure 4:**
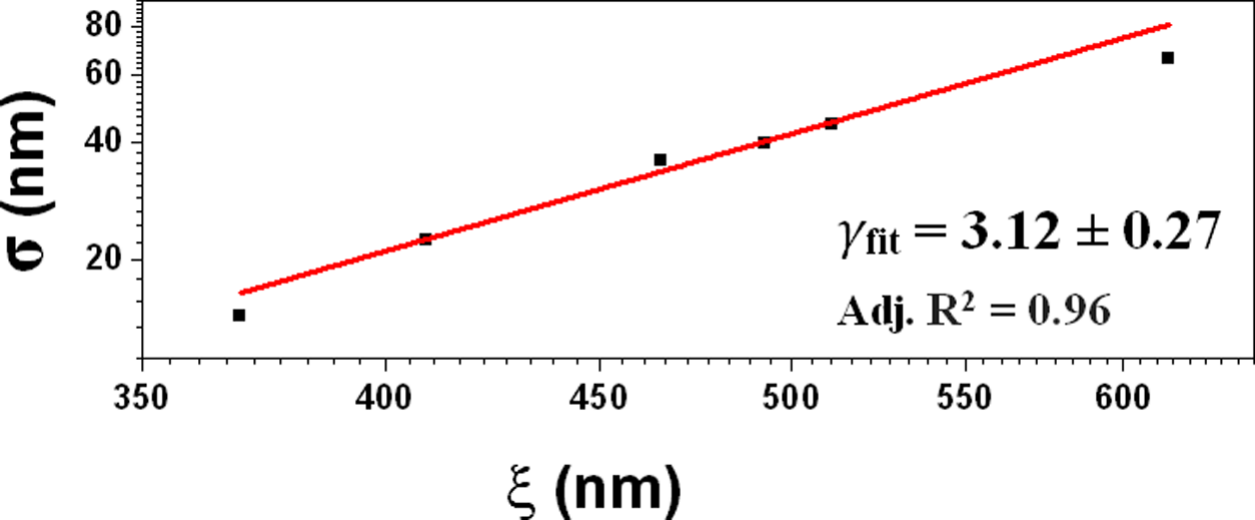
log–log plot of the RMS roughness (σ) versus the lateral correlation length (ξ) calculated considering all of the films. The γ_fit_ value extracted through σ ∼ ξ^γ^ is also shown, with the adjusted *R*^2^.

To characterize more precisely the dynamics of the growth, the dependence of the parameter σ on ξ, following the power law σ ∼ ξ^γ^, can be considered. Interestingly, the exponent γ is related to the competition between the vertical and the lateral growth of the islands, being γ > 1 when the vertical growth is faster with respect to the lateral one.^51^ This is the case of the system investigated here since the fit of σ versus ξ gives γ_fit_ = 3.12 ± 0.27 (within experimental errors, Figure 4).

This behavior is compatible with the presence of step-edge barriers, such as the Ehrlich–Schwoebel (ES) barrier,^52−54^ which promotes a more vertical growth and is also consistent with the preferential growth at the edges of the islands during film evolution. It should be finally noted that γ theoretically corresponds to the ratio β/(1/*z*) = β*z*, which gives γ = 3.00 ± 0.28, fully compatible with the value of γ_fit_ (within the respective errors).

In summary, from the AFM monitoring and the HHCF analysis, some characteristics of the growth of NiTPP on HOPG have been obtained and quantified: the rather high value of α (0.85) describing a locally smooth surface; the value of β (0.44) compatible with a roughening process; and the presence of a lateral growth given by the value of 1/*z* (0.15), together with a significant and faster vertical growth supported by γ > 1.

Beyond the quantitative description of NiTPP growth on HOPG, the main outcome of the present study is the full assessment of the HHCF analysis of films subjected to post-growth evolution, considering the latter as an integral part of the growth itself. In other words, this work clearly shows that the growth process starts with film deposition and ends when the film morphology reaches the “steady state”. Therefore, the HHCF analysis is reliable only when applied to AFM images collected at the end of the whole process, as indeed shown in this study. To the best of the authors’ knowledge, the approach adopted here for describing the growth in the case of post-growth evolving systems has not been reported before. Actually, in most cases, the post-growth evolution is not contemplated possibly because of the high stability of the films. When observed, it is not considered for the extraction of the scaling exponents from the HHCF analysis.^12^

## Conclusions

The growth of NiTPP thin films deposited on HOPG is studied by means of AFM and through the HHCF analysis. All of the films showed post-growth evolution in ambient conditions at room temperature to be studied for a proper understanding of the material and to be taken into account for their integration into devices.^55−59^ The set of scaling exponents obtained from a careful use of the HHCF analysis suggests that the growth is driven by an interplay between diffusion and step-edge barrier effect, in agreement with the observed Ostwald-like ripening. This work attests to the reliability of the HHCF analysis also in the case of films displaying post-growth evolution, provided that such an evolution is considered an integral part of the growth process itself. These findings open new perspectives in the understanding of the growth and properties of other organic or inorganic films affected by post-growth phenomena.

## Supporting Information Available

Supplementary AFM images, together with further graphs illustrating the HHCF analysis done on both the samples as extracted from the vacuum chamber and in their “steady state” (PDF)

## References

[ref1] Anthony J. E.; Facchetti A.; Heeney M.; Marder S. R.; Zhan X. N-Type Organic Semiconductors in Organic Electronics. Adv. Mater. 2010, 22, 3876–3892. 10.1002/adma.200903628. 20715063

[ref2] Reese C.; Roberts M.; Ling M.-m.; Bao Z. Organic Thin Film Transistors. Mater. Today 2004, 7, 20–27. 10.1016/S1369-7021(04)00398-0.

[ref3] Yan D.; Wang H.; Du B. Introduction to Organic Semiconductor Heterojunctions; John Wiley & Sons, 2010; pp 155–205.

[ref4] Siegrist T.; Kloc C.; Schön J. H.; Batlogg B.; Haddon R. C.; Berg S.; Thomas G. A. Enhanced Physical Properties in a Pentacene Polymorph. Adv. Carbohydr. Chem. Biochem. 2001, 40, 1732–1736. 10.1002/1521-3757(20010504)113:9<1782::AID-ANGE17820>3.0.CO;2-Q. 11353494

[ref5] Ahmad F. N.; Wijaya Y. P.; Mohamad K. A.; Nayan N.; Megat Hasnan M. M. I.; Alias A.; Ghosh B. K. Morphological, Structural and Electrical Properties of Pentacene Thin Films Grown via Thermal Evaporation Technique. Bull. Electr. Eng. Inf. 2021, 10, 1291–1299. 10.11591/eei.v10i3.3029.

[ref6] Thankaraj Salammal S.; Chen J.; Ullah F.; Chen H. Effects of Material Morphology on the Performance of Organic Electronics. J. Inorg. Organomet. Polym. Mater. 2015, 25, 12–26. 10.1007/s10904-014-0107-z.

[ref7] Sassella A.; Campione M.; Borghesi A. Organic Epitaxy. Riv. Nuovo Cimento 2008, 31, 457–490. 10.1393/ncr/i2008-10035-y.

[ref8] Schreiber F. Organic Molecular Beam Deposition: Growth Studies beyond the First Monolayer. Phys. Status Solidi A 2004, 201, 1037–1054. 10.1002/pssa.200404334.

[ref9] Forrest S. R. Ultrathin Organic Films Grown by Organic Molecular Beam Deposition and Related Techniques. Chem. Rev. 1997, 97, 1793–1896. 10.1021/cr941014o. 11848893

[ref10] Trabattoni S.; Raimondo L.; Campione M.; Braga D.; Holmberg V. C.; Norris D. J.; Moret M.; Ciavatti A.; Fraboni B.; Sassella A. Substrate Selection for Full Exploitation of Organic Semiconductor Films: Epitaxial Rubrene on β-Alanine Single Crystals. Adv. Mater. Interfaces 2015, 2, 1500423 10.1002/admi.201500423.

[ref11] Kowarik S.; Gerlach A.; Schreiber F. Organic Molecular Beam Deposition: Fundamentals, Growth Dynamics, and in Situ Studies. J. Phys. Condens. Matter. 2008, 20, 184005 10.1088/0953-8984/20/18/184005.

[ref12] Chiarella F.; Perroni C. A.; Chianese F.; Barra M.; de Luca G. M.; Cataudella V.; Cassinese A. Post-Deposition Wetting and Instabilities in Organic Thin Films by Supersonic Molecular Beam Deposition. Sci. Rep. 2018, 8, 12015 10.1038/s41598-018-30567-7. 30104704 PMC6089966

[ref13] Krause B.; Dürr A. C.; Schreiber F.; Dosch H.; Seeck O. H. Thermal Stability and Partial Dewetting of Crystalline Organic Thin Films: 3,4,9,10-Perylenetetracarboxylic Dianhydride on Ag(111). J. Chem. Phys. 2003, 119, 3429–3435. 10.1063/1.1589471.

[ref14] Balzer F.; Schiek M.; Osadnik A.; Wallmann I.; Parisi J.; Rubahn H. G.; Lützen A. Substrate Steered Crystallization of Naphthyl End-Capped Oligothiophenes into Nanofibers: The Influence of Methoxy-Functionalization. Phys. Chem. Chem. Phys. 2014, 16, 5747–5754. 10.1039/c3cp53881h. 24531698

[ref15] Scherwitzl B.; Lukesch W.; Hirzer A.; Albering J.; Leising G.; Resel R.; Winkler A. Initial Steps of Rubicene Film Growth on Silicon Dioxide. J. Phys. Chem. C 2013, 117, 4115–4123. 10.1021/jp3122598. PMC3589099 23476720

[ref16] Ribič P. R.; Bratina G. Ripening of Rubrene Islands. J. Phys. Chem. C 2007, 111, 18558–18562. 10.1021/jp077291j.

[ref17] Raimondo L.; Trabattoni S.; Sassella A. Control of Post-Growth Processes for the Selection of Metallo-Tetraphenylporphyrin Nanowires. Phys. Chem. Chem. Phys. 2019, 21, 8482–8488. 10.1039/C8CP07747A. 30957123

[ref18] Topple J. M.; Burke S. A.; Fostner S.; Grütter P. Thin Film Evolution: Dewetting Dynamics of a Bimodal Molecular System. Phys. Rev. B 2009, 79, 205414 10.1103/PhysRevB.79.205414.

[ref19] Dienel T.; Loppacher C.; Mannsfeld S. C. B.; Forker R.; Fritz T. Growth-Mode-Induced Narrowing of Optical Spectra of an Organic Adlayer. Adv. Mater. 2008, 20, 959–963. 10.1002/adma.200701684.

[ref20] Shi J.; Qin X. R. Nucleation and Growth of Tetracene Films on Silicon Oxide. Phys. Rev. B 2008, 78, 115412–115416. 10.1103/PhysRevB.78.115412.

[ref21] Krause B.; Dürr A. C.; Schreiber F.; Dosch H.; Seeck O. H. Late Growth Stages and Post-Growth Diffusion in Organic Epitaxy: PTCDA on Ag(111). Surf. Sci. 2004, 572, 385–395. 10.1016/j.susc.2004.09.011.

[ref22] Kowarik S.; Gerlach A.; Sellner S.; Cavalcanti L.; Schreiber F. Dewetting of an Organic Semiconductor Thin Film Observed in Real-Time. Adv. Eng. Mater. 2009, 11, 291–294. 10.1002/adem.200800289.

[ref23] Virkar A. A.; Mannsfeld S. C. B.; Bao Z. Energetics and Stability of Pentacene Thin Films on Amorphous and Crystalline Octadecylsilane Modified Surfaces. J. Mater. Chem. 2010, 20, 2664–2671. 10.1039/b921767c.

[ref24] Amassian A.; Pozdin V. A.; Desai T. V.; Hong S.; Woll A. R.; Ferguson J. D.; Brock J. D.; Malliaras G. G.; Engstrom J. R. Post-Deposition Reorganization of Pentacene Films Deposited on Low-Energy Surfaces. J. Mater. Chem. 2009, 19, 5580–5592. 10.1039/b907947e.

[ref25] Chiodini S.; D’Avino G.; Muccioli L.; Bartolini L.; Gentili D.; Toffanin S.; Albonetti C. Self-Organization of Complete Organic Monolayers via Sequential Post-Deposition Annealing. Prog. Org. Coat. 2020, 138, 105408–105421. 10.1016/j.porgcoat.2019.105408.

[ref26] Bartolini L.; Malferrari M.; Lugli F.; Zerbetto F.; Paolucci F.; Pelicci P. G.; Albonetti C.; Rapino S. Interaction of Single Cells with 2D Organic Monolayers: A Scanning Electrochemical Microscopy Study. ChemElectroChem 2018, 5, 2975–2981. 10.1002/celc.201800731.

[ref27] Sassella A.; Raimondo L.; Campione M.; Borghesi A. Patterned Growth of Crystalline Organic Heterostructures. Adv. Mater. 2013, 25, 2804–2808. 10.1002/adma.201300462. 23568542

[ref28] Gentili D.; Foschi G.; Valle F.; Cavallini M.; Biscarini F. Applications of Dewetting in Micro and Nanotechnology. Chem. Soc. Rev. 2012, 41, 4430–4443. 10.1039/c2cs35040h. 22491348

[ref29] Itoh T.; Yamauchi N. Surface Morphology Characterization of Pentacene Thin Film and Its Substrate with Under-Layers by Power Spectral Density Using Fast Fourier Transform Algorithms. Appl. Surf. Sci. 2007, 253, 6196–6202. 10.1016/j.apsusc.2007.01.056.

[ref30] Fekete L.; Kůsová K.; Petrák V.; Kratochvílová I. AFM Topographies of Densely Packed Nanoparticles: A Quick Way to Determine the Lateral Size Distribution by Autocorrelation Function Analysis. J. Nanopart. Res. 2012, 14, 1062 10.1007/s11051-012-1062-7.

[ref31] Dürr A. C.; Schreiber F.; Ritley K. A.; Kruppa V.; Krug J.; Dosch H.; Struth B. Rapid Roughening in Thin Film Growth of an Organic Semiconductor (Diindenoperylene). Phys. Rev. Lett. 2003, 90, 016104 10.1103/PhysRevLett.90.016104. 12570630

[ref32] Gedda M.; Subbarao N. V. V.; Goswami D. K. Local Diffusion Induced Roughening in Cobalt Phthalocyanine Thin Film Growth. Langmuir 2014, 30, 8735–8740. 10.1021/la502108a. 24992503

[ref33] Kardar M.; Parisi G.; Zhang Y.-C. Dynamic Scaling of Growing Interfaces. Phys. Rev. Lett. 1986, 56, 889–892. 10.1103/PhysRevLett.56.889. 10033312

[ref34] Krug J. Origins of Scale Invariance in Growth Processes. Adv. Phys. 1997, 46, 139–282. 10.1080/00018739700101498.

[ref35] Tang C.; Alexander S.; Bruinsma R.; Shaw B. E. Scaling Theory for the Growth of Amorphous Films. Phys. Rev. Lett. 1990, 64, 772–775. 10.1103/PhysRevLett.64.772. 10042074

[ref36] Pelliccione M.; Lu T.-M. Evolution of Thin Film Morphology: Modeling and Simulations; Springer-Verlag, 2008; pp 29–56.

[ref37] Gredig T.; Silverstein E. A.; Byrne M. P. Height-Height Correlation Function to Determine Grain Size in Iron Phthalocyanine Thin Films. J. Phys.: Conf. Ser. 2013, 417, 012069 10.1088/1742-6596/417/1/012069.

[ref38] Obaidulla Sk. Md.; Giri P. K. Surface Roughening and Scaling Behavior of Vacuum-Deposited SnCl _2_ Pc Organic Thin Films on Different Substrates. Appl. Phys. Lett. 2015, 107, 221910–221915. 10.1063/1.4936937.

[ref39] Zhang Y.; Barrena E.; Zhang X.; Turak A.; Maye F.; Dosch H. New Insight into the Role of the Interfacial Molecular Structure on Growth and Scaling in Organic Heterostructures. J. Phys. Chem. C 2010, 114, 13752–13758. 10.1021/jp103841t.

[ref40] Valle F.; Brucale M.; Chiodini S.; Bystrenova E.; Albonetti C. Nanoscale Morphological Analysis of Soft Matter Aggregates with Fractal Dimension Ranging from 1 to 3. Micron 2017, 100, 60–72. 10.1016/j.micron.2017.04.013. 28514702

[ref41] Gamboa M.; Campos M.; Torres L. A. Study of the Stability of 5,10,15,20-Tetraphenylporphine (TPP) and Metalloporphyrins NiTPP, CoTPP, CuTPP, and ZnTPP by Differential Scanning Calorimetry and Thermogravimetry. J. Chem. Thermodyn. 2010, 42, 666–674. 10.1016/j.jct.2009.12.007.

[ref42] Albani G.; Calloni A.; Picone A.; Brambilla A.; Capra M.; Lodesani A.; Duò L.; Finazzi M.; Ciccacci F.; Bussetti G. An In-Depth Assessment of the Electronic and Magnetic Properties of a Highly Ordered Hybrid Interface: The Case of Nickel Tetra-Phenyl-Porphyrins on Fe(001)-p(1 × 1)O. Micromachine 2021, 12, 191 10.3390/mi12020191. PMC7918924 33668500

[ref43] Nawar A. M.; Abdel-Khalek H.; Mohamed El-Nahass M.; Abd El-Khalek H. M.; El-Nahass M. M. Dielectric and Electric Modulus Studies on Ni (II) Tetraphenyl Porphyrin Thin Films. Org. Opto-Elect. 2015, 1, 25–38.

[ref44] El-Nahass M. M.; Abd El-Khalek H. M.; Nawar A. M. Structural and Optical Characterizations of Ni (II) Tetraphenyl Porphyrin Thin Films. Eur. Phys. J.: Appl. Phys. 2012, 57, 30201–30214. 10.1051/epjap/2012110280.

[ref45] Horcas I.; Fernández R.; Gómez-Rodríguez J. M.; Colchero J.; Gómez-Herrero J.; Baro A. M. WSXM: A Software for Scanning Probe Microscopy and a Tool for Nanotechnology. Rev. Sci. Instrum. 2007, 78, 013705–013708. 10.1063/1.2432410. 17503926

[ref46] Nečas D.; Klapetek P. Gwyddion: An Open-Source Software for SPM Data Analysis. Cent. Eur. J. Phys. 2012, 10, 181–188. 10.2478/s11534-011-0096-2.

[ref47] Tang Y.; Wang Y.; Wang X.; Xun S.; Mei C.; Wang L.; Yan D. AFM Observations of Phase Transitions in Molecularly Thin Films of a Three-Ring Bent-Core Compound. J Phys. Chem. B 2005, 109, 8813–8819. 10.1021/jp045427x. 16852047

[ref48] Pelliccione M.; Karabacak T.; Gaire C.; Wang G. C.; Lu T. M. Mound Formation in Surface Growth under Shadowing. Phys. Rev. B 2006, 74, 125420 10.1103/PhysRevB.74.125420. 16712101

[ref49] Agrawal A.; Tchoe Y.; Kim H.; Park J. Y. Qualitative Analysis of Growth Mechanism of Polycrystalline InAs Thin Films Grown by Molecular Beam Epitaxy. Appl. Surf. Sci. 2018, 462, 81–85. 10.1016/j.apsusc.2018.08.076.

[ref50] Chiodini S.; Straub A.; Donati S.; Albonetti C.; Borgatti F.; Stoliar P.; Murgia M.; Biscarini F. Morphological Transitions in Organic Ultrathin Film Growth Imaged by in Situ Step-by-Step Atomic Force Microscopy. J. Phys. Chem. C 2020, 124, 14030–14042. 10.1021/acs.jpcc.0c03279.

[ref51] Kesarwani R.; Dey P. P.; Khare A. Correlation between Surface Scaling Behavior and Surface Plasmon Resonance Properties of Semitransparent Nanostructured Cu Thin Films Deposited via PLD. RSC Adv. 2019, 9, 7967–7974. 10.1039/C9RA00194H. 35521153 PMC9061400

[ref52] Ehrlich G.; Hudda F. G. Atomic View of Surface Self-Diffusion: Tungsten on Tungsten. J. Chem. Phys. 1966, 44, 1039–1049. 10.1063/1.1726787.

[ref53] Schwoebel R. L. Step Motion on Crystal Surfaces. II. J. Appl. Phys. 1969, 40, 614–618. 10.1063/1.1657442.

[ref54] Hlawacek G.; Puschnig P.; Frank P.; Winkler A.; Ambrosch-Draxl C.; Teichert C. Characterization of Step-Edge Barriers in Organic Thin-Film Growth. Science 2008, 321, 108–111. 10.1126/science.1159455. 18599783

[ref55] Rivera M.; Rivera J. M.; Amelines-Sarria O.; Martínez-García M. Evaporated Porphyrin Films as Nitrogen Dioxide Gas Sensors. Bull. Mater. Sci. 2019, 42, 50 10.1007/s12034-019-1735-2.

[ref56] Magna G.; Mandoj F.; Stefanelli M.; Pomarico G.; Monti D.; di Natale C.; Paolesse R.; Nardis S. Recent Advances in Chemical Sensors Using Porphyrin-Carbon Nanostructure Hybrid Materials. Nanomaterials 2021, 11, 997 10.3390/nano11040997. 33924607 PMC8069093

[ref57] Park J. M.; Lee J. H.; Jang W. D. Applications of Porphyrins in Emerging Energy Conversion Technologies. Coord. Chem. Rev. 2020, 407, 213157 10.1016/j.ccr.2019.213157.

[ref58] Lin V. S.-Y.; DiMagno S. G.; Therien M. J. Highly Conjugated, Acetylenyl Porphyrins: New Models for Light-Harvesting Antenna Systems. Science 1994, 246, 1105–1111. 10.1126/science.8178169. 8178169

[ref59] Mandal T.; Das S.; de Sarkar S. Nickel(II) Tetraphenylporphyrin as an Efficient Photocatalyst Featuring Visible Light Promoted Dual Redox Activities. Adv. Synth. Catal. 2019, 361, 3200–3209. 10.1002/adsc.201801737.

