# Supplementary material for: Post-Growth Dynamics and Growth Modeling of Organic Semiconductor Thin Films
Source: Langmuir. 2023 Feb 22;39(9):3266–72. doi: 10.1021/acs.langmuir.2c03066 (PMC9996818; doi:10.1021/acs.langmuir.2c03066)
Supplement: Supplementary file 1 — la2c03066_si_001.pdf [file la2c03066_si_001.pdf]

Supporting information

Post-growth dynamics and growth modelling of organic semiconductor thin films

Alice Pancaldi,*† Luisa Raimondo,† Alessandro Minotto,† Adele Sassella**†

† Department of Materials Science, University of Milano-Bicocca, via R. Cozzi 55, 20125 Milano (Italy)
Corresponding authors:
*a.pancaldi@campus.unimib.it
**adele.sassella@unimib.it

1) Film morphology and AFM monitoring

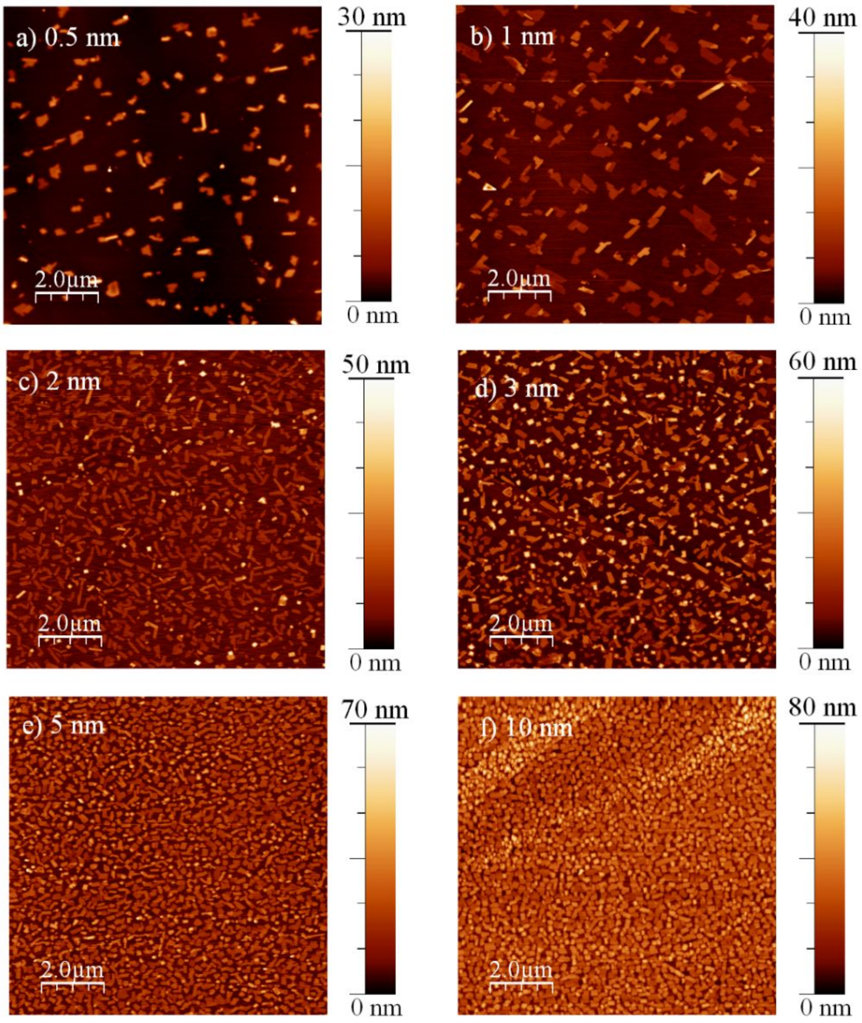

Figure S1. 10 × 10 μm^2 AFM height images of all the samples taken at t_a = 1 h, except for image in d) which was taken at t_a = 2.5 h. The thickness of each film is reported on each image.

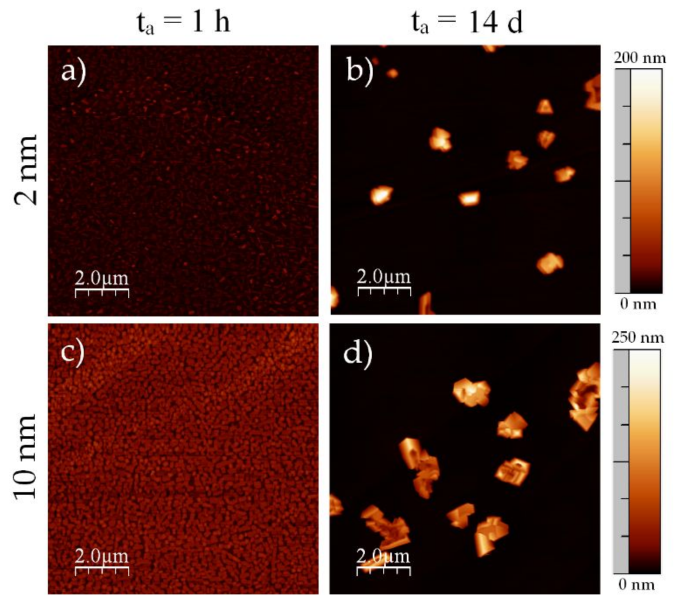

Figure S2. Example of the evolution in air of two films with different nominal thickness. a-b) 2 nm, taken at t_a = 1 h and 10 d, respectively; c-d) 10 nm, taken at t_a = 1 h and 14 d, respectively. The height scale on the right refers to all the images in the same row.

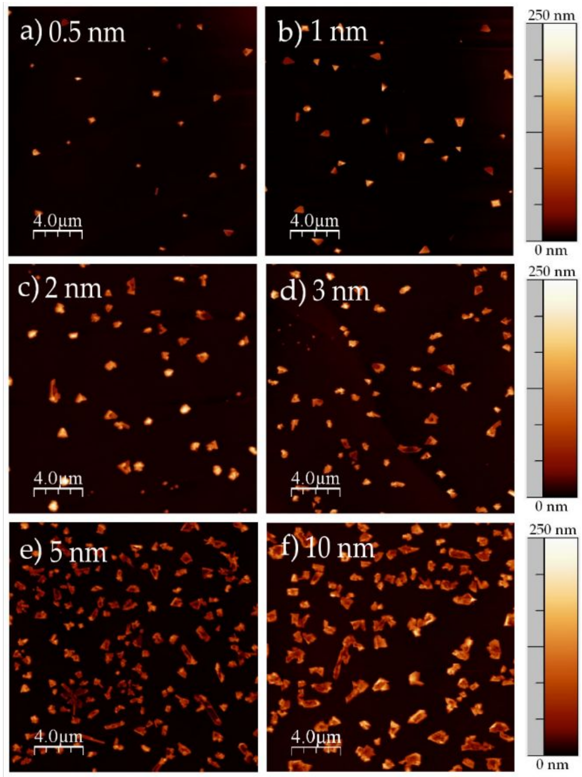

Figure S3. 20 × 20 μm^2 AFM images of stable films (t_a up to 14 days). Film obtained keeping fixed the deposition rate, and varying only the deposition time. The values of the thickness are reported in the Figure.

2) Analysis and modelling

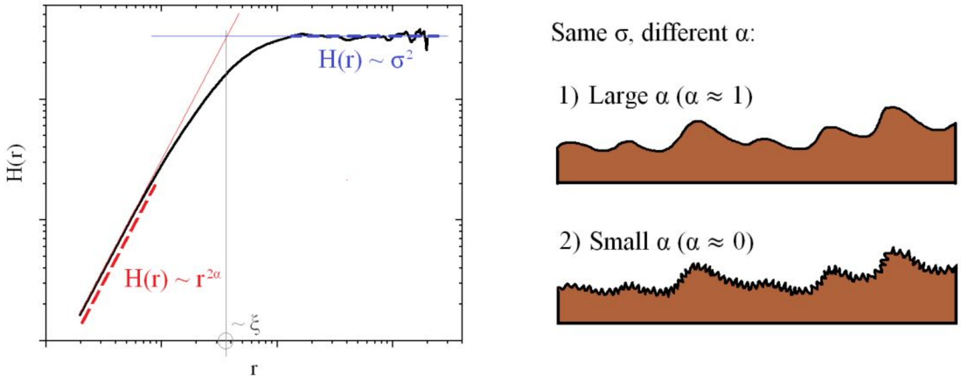

Figure S4. A picture showing the meaning of different long-range roughness (σ) and the short-range roughness (α) values, and their representation in a typical HHCF curve. On the left: the HHCF and the visual representation of the parameters σ and ξ. On the right: the meaning of the different values of the exponent α, keeping σ fixed.

Fast Fourier transform on the images

In support of the choice to model the observed morphology with Eq. 3, the 2D fast Fourier transform (FTT) is carried out on all the AFM images: a disc-like behavior (see Figure S5) is found, typical of mounded morphology.[1] The use of Eq. 3 for the HHCF analysis is, therefore, fully justified also in case of island-like morphology, as the one observed in our films.

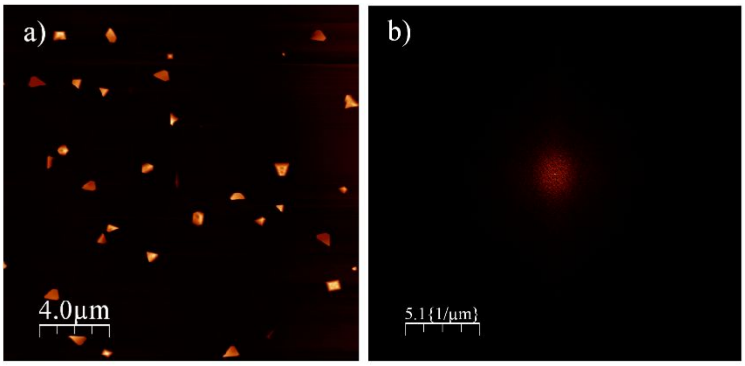

Figure S5. (a) An example of AFM image of 1 nm sample and (b) its relative fast Fourier transform.

Analysis on the images taken at t_a~ 0

In other papers reporting a similar analysis on organic thin films, it's not always specified whether the analysis it's applied to the images acquired as soon as the sample is extracted from the deposition chamber or after hours or days in ambient conditions. This detail it's relevant in this case because our films face a significant post-growth evolution, so the application of this analysis would lead to different curves depending on when the AFM images were acquired. Given this, we decided to exploit images of the films acquired as soon as the evolution stops and reaches a steady-state configuration.

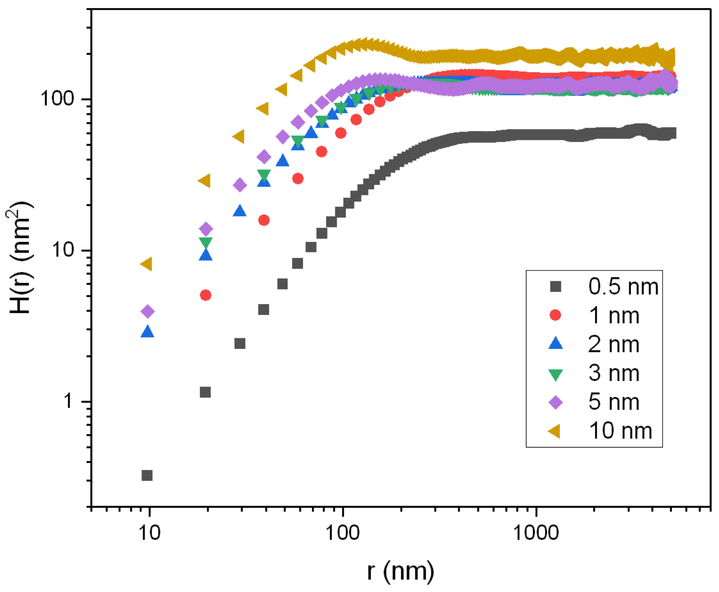

Figure S6. HHCF curves obtained analyzing the images at the shortest t_a (taken within 30 minutes since the extraction from the vacuum chamber).

The HHCF curves obtained from the images collected right after extraction do not follow the expected trend when comparing films with different thickness.[2] Namely, referring to Figure S4, one would expect the σ value (i.e., the asymptotic value of $H(r)$ for $r \gg \xi$) to increase with t. Therefore, applying the HHCF analysis to these data means comparing different stages of the growth, and is thereby meaningless.

HHCF data fits on steady-state films:

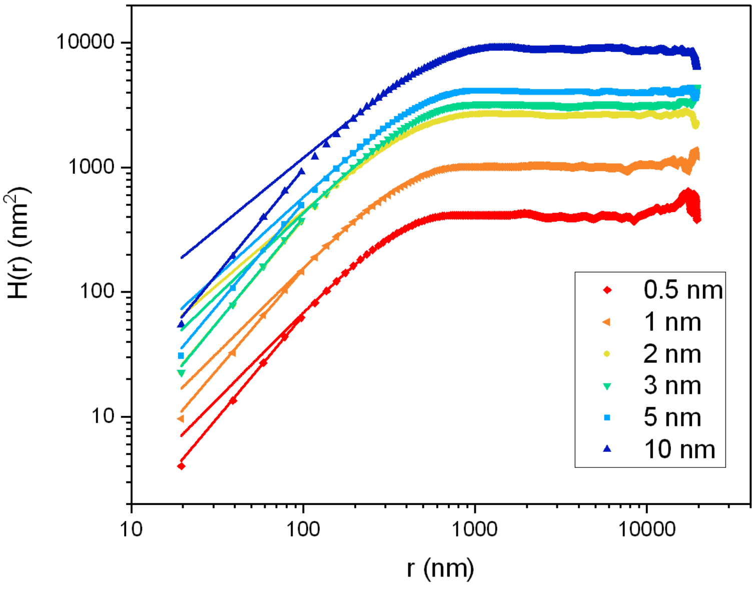

Figure S7. HHCF data plots and fits to Eqs. 3 and 4, in the total range of r and for $r \ll \xi$ respectively.

References

(1) Obaidulla, Sk. Md.; Giri, P. K. Surface Roughening and Scaling Behavior of Vacuum-Deposited $SnCl_2$ Pc Organic Thin Films on Different Substrates. *Appl. Phys. Lett.* **2015**, *107*, 221910–221915.

(2) Pelliccione, M.; Lu, T.-M. *Evolution of Thin Film Morphology: Modeling and Simulations*; Springer-Verlag: New York, 2008.
